# Proprotein convertase subtilisin/kexin type 9 (PCSK9) levels are not associated with severity of liver disease and are inversely related to cholesterol in a cohort of thirty eight patients with liver cirrhosis

**DOI:** 10.1186/s12944-021-01431-x

**Published:** 2021-01-18

**Authors:** Susanne Feder, Reiner Wiest, Thomas S. Weiss, Charalampos Aslanidis, Doris Schacherer, Sabrina Krautbauer, Gerhard Liebisch, Christa Buechler

**Affiliations:** 1 Department of Internal Medicine I, Regensburg University Hospital, D-93042 Regensburg, Germany; 2 Department of Visceral Surgery and Medicine, University Inselspital, Bern, Switzerland; 3 Children’s University Hospital (KUNO), Regensburg University Hospital, Regensburg, Germany; 4 Institute of Clinical Chemistry and Laboratory Medicine, Regensburg University Hospital, Regensburg, Germany

**Keywords:** Ceramide, Sphingomyelin, Chemerin, Model for end-stage liver disease score, Ascites, Varices, Alcoholic, Hepatitis C

## Abstract

**Background:**

Proprotein convertase subtilisin/kexin type 9 (PCSK9) is of particular importance in cholesterol metabolism with high levels contributing to hypercholesterolemia. Cholesterol and sphingolipids are low in patients with liver cirrhosis. Purpose of this study was to find associations of plasma PCSK9 with circulating cholesterol and sphingolipid species and measures of liver disease severity in patients with liver cirrhosis.

**Methods:**

PCSK9 protein levels were determined by ELISA in systemic vein (SVP), hepatic vein (HVP) and portal vein plasma of patients with mostly alcoholic liver cirrhosis. PCSK9 and LDL-receptor protein expression were analysed in cirrhotic and non-cirrhotic liver tissues.

**Results:**

Serum PCSK9 was reduced in patients with liver cirrhosis in comparison to non-cirrhotic patients. In liver cirrhosis, plasma PCSK9 was not correlated with Child-Pugh score, Model for End-Stage Liver Disease score, bilirubin or aminotransferases. A negative association of SVP PCSK9 with albumin existed. PCSK9 protein in the liver did not change with fibrosis stage and was even positively correlated with LDL-receptor protein levels. Ascites volume and variceal size were not related to PCSK9 levels. Along the same line, transjugular intrahepatic shunt to lower portal pressure did not affect PCSK9 concentrations in the three blood compartments. Serum cholesterol, sphingomyelin and ceramide levels did not correlate with PCSK9. Stratifying patients by high versus low PCSK9 levels using the median as cut-off, several cholesteryl ester species were even low in the subgroup with high PCSK9 levels. A few sphingomyelin species were also reduced in the patients with PCSK9 levels above the median. PCSK9 is highly expressed in the liver but systemic, portal and hepatic vein levels were similar. PCSK9 was not correlated with the inflammatory proteins C-reactive protein, IL-6, galectin-3, resistin or pentraxin 3. Of note, HVP PCSK9 was positively associated with HVP chemerin and negatively with HVP adiponectin levels.

**Conclusions:**

In the cohort of patients with liver cirrhosis mostly secondary to alcohol consumption high PCSK9 was associated with low levels of certain cholesteryl ester and sphingomyelin species. Positive correlations of PCSK9 and LDL-receptor protein in the liver of patients with chronic liver injury are consistent with these findings.

## Introduction

Liver cirrhosis is the stage of chronic liver diseases with irreversible organ damage. Chronic infections with hepatitis B or C virus, alcohol abuse and non-alcoholic steatohepatitis (NASH) are the most common causes of liver cirrhosis [1]. Diagnosis of liver cirrhosis is usually based on physical examination, blood tests and sonography and liver biopsy is in general not necessary [1]. Liver dysfunction and portal hypertension in liver cirrhosis can lead to serious complications, and the most common are varices, ascites and hepatic encephalopathy [2]. The Child-Pugh score is used to assess the severity of liver dysfunction in the clinic. This score includes ascites, hepatic encephalopathy, total bilirubin, albumin, prothrombin time or international normalized ratio (INR) [3]. Model for end-stage liver disease (MELD) score was first approved for the prediction of the survival of patients undergoing transjugular intrahepatic portosystemic shunts (TIPS) [3]. The MELD score includes INR, total bilirubin and creatinine and nowadays is used for the prioritization of liver transplantation candidates [3].

The liver is the central organ in lipid metabolism, and abnormalities of lipoproteins are common in patients with liver cirrhosis. Low density (LDL) and high density lipoprotein (HDL) were reduced in patients with liver cirrhosis in comparison to liver-healthy controls and were lowest in patients with decompensated disease [4–6]. Lipoprotein particles contain cholesterol [7] and serum cholesterol was accordingly low in liver cirrhosis [8]. Cholesterol concentration of LDL is about 4-fold higher than levels in HDL particles [7] and both lipoproteins declined in liver cirrhosis patients [9].

Proprotein convertase subtilisin/kexin type 9 (PCSK9) plays a key role in cholesterol metabolism. PCSK9 promotes the lysosomal degradation of the LDL-receptor and increases plasma LDL cholesterol [10, 11]. Targeting PCSK9 is a promising, but costly approach, to treat hypercholesterolemia [10, 11].

Lipid metabolism is disturbed in patients with NASH suggesting a role of PCSK9 herein. In morbidly obese patients hepatic PCSK9 protein was reduced in the patients with fatty liver whereas associations with hepatic inflammation and hepatocyte ballooning did not exist [12]. Although positive correlations of circulating PCSK9 with hepatic steatosis and markers of liver injury existed in some cohorts [12, 13] such associations could not be identified in other studies [14].

The function of PCSK9 beyond its role in cholesterol metabolism is not well understood [10]. PCSK9 positively correlated with C-reactive protein (CRP) and white blood cell number indicating a role in the immune response [15]. In fact, PCSK9 production was induced by inflammatory stimuli and by itself was a proinflammatory factor [10, 16, 17]. PCSK9 inhibition thus suppressed nuclear factor kappa B activation and attenuated inflammation in experimental colitis [18]. PCSK9 deficient mice challenged with lipopolysaccharide (LPS) had reduced circulating levels of proinflammatory cytokines [19]. The effects of PCSK9 blockage were abrogated in LDL-receptor deficient mice. This suggests that bacterial lipids such as LPS were cleared via the LDL-receptor. Interestingly, LPS response was also attenuated in humans with PCSK9 loss-of-function mutations [19].

Dysregulation of the immune response in patients with liver cirrhosis contributes to fibrosis [20]. Inflammatory biomarkers were related to patients’ survival and the prognostic value of IL-6 for mortality was comparable with the MELD score [21]. Blockage of PCSK9 enhanced LPS clearance and ameliorated systemic and hepatic inflammation in a rat model of liver cirrhosis [22]. PCSK9 inhibition moreover was effective in a rodent model of alcoholic liver disease, and improved hepatic inflammation [23]. Even though these experimental data suggest PCSK9 inhibitors as valuable options in patients with liver cirrhosis, low serum PCSK9 was associated with a higher mortality in patients with end-stage liver disease [24]. In this cohort, serum PCSK9 was negatively correlated with the MELD score, bilirubin and INR [24]. A separate study reported on increased serum PCSK9 values in patients with liver cirrhosis compared to non-cirrhotic patients with chronic liver disease. This was in line with higher levels of PCSK9 protein in the cirrhotic liver [25]. In patients with liver cirrhosis, serum cholesterol and PCSK9 concentrations were not correlated [24].

In human carriers of a PCSK9 loss-of-function variant (R46L), distinct cholesteryl ester and sphingolipid species were reduced as compared to carriers of the major allele [26]. LDL-cholesterol did not significantly differ between these two groups and variations in serum sphingolipid composition could be a more sensitive markers of PCSK9 activity [26]. Lipoproteins contain sphingolipids and about 60% of serum ceramide and 30 to 50% of serum sphingomyelin are part of LDL particles. HDL carries about 25% of serum ceramide and about 40% of serum sphingomyelin [7, 27]. Enhancing the hepatic uptake of LDL most likely lowers serum cholesterol and other lipids including ceramide and sphingomyelin. A general decrease of lipids was indeed detected in LDL particles of patients with coronary heart disease who were treated with a PCSK9 inhibiting antibody [28].

In this study we hypothesized that systemic PCSK9 levels correlate with cholesterol and / or sphingolipid species and possibly parameters of inflammation in patients with liver cirrhosis. Regarding that PCSK9 is highly expressed in the liver [29], we postulated a negative association of hepatic vein PCSK9 levels with residual liver function.

## Materials and methods

### Patients

Thirty eight patients with liver cirrhosis were included in the study. Cirrhosis was diagnosed by physical examination, sonography and laboratory values. Details of the cohort are listed in Table 1. The causes for liver cirrhosis were alcohol abuse in 31 patients, chronic hepatitis C (HCV) infection in 3 patients and of different etiologies in 4 patients. Transjugular intrahepatic portosystemic shunt **(**TIPS) (Viatorr-Stent, Putzbrunn, Germany) implantation was done in the morning in the fasted patient [30]. TIPS was inserted in 12 patients with variceal bleeding, in 1 patient with hepatorenal syndrome, in 24 patients with refractory ascites and in 1 patient where the reason for stent insertion was not documented. Ascites volume was defined as: little = ascites only detected by ultrasound; massive = extensive and bulging ascites; modest = in between little and massive. Small varices disappeared in endoscopy during air insufflation and large varices did not.

**Table 1:** Patient demographics and laboratory parameters (C-reactive protein, CRP; alanine aminotransferase, ALT; aspartate aminotransferase, AST). Median values and range of the values are shown. Superscript digits were added when the respective information was not available for the whole study group and correspond to the number of patients where these data were documented. Not applicable, na; not determined, nd; *** *P* < 0.001 for comparison of serum parameters from cirrhotic and non-cirrhotic patients

	Cirrhosis Patients Plasma	Cirrhosis Patients Serum	Non-cirrhosis Patients Serum
Number	38	26	55
Sex (female/male)	9/29	5/21	23/32
Age (years)	53 (26–81)	49 (40–81)	58 (21–80)
Child-Pugh score A/B/C/nd	10/13/15/0	8/7/7/4	na
MELD score	9 (6–21)	8 (6–21) ^22^	na
Ascites: no / little / modest / massive /nd	4/11/4/19/0	6/9/2/8/1	na
Variceal size: no / small / large / nd	6/7/25/0	5/4/16/1	na
C-reactive protein (mg/L)	14.4 (1.0–53.5) ^35^	13.9 (2.0–46.7) ^20^	nd
Fibrinogen (mg/dL)	263 (114–520) ^37^	349 (151–515) ^22^	nd
Antithrombin 3 (%)	63.7 (23.6–100.0) ^34^	65 (23.6–92.8) ^21^	nd
Factor V (%)	59 (17–137) ^25^	61 (17–127) ^20^	nd
ALT (U/l)	38 (4–108)	38 (7–108)	32 (16–288) ^54^
AST (U/l)	30.0 (2–84)	29 (2–73)	24 (12–256) ^54^
Albumin (g/L)	31.2 (1.6–47.0)	31.5 (1.6–4.1)	46.8 (31.6–55.6) ***
Bilirubin (mg/dL)	1.2 (0.3–8.2)	1.1 (0.4–3.7)	0.5 (0.2–2.8) ^53 ***^
Quick prothrombin time (%)	72 (28–100)	76 (44–100) ^22^	nd
Creatinine (mg/dL)	1.1 (0.5–4.5)	1.0 (0.6–4.5) ^22^	nd
LDL (mg/dL)	60 (6-128)	35 (17–81)	112 (44–340)^52^ ***

During TIPS, EDTA plasma was obtained from the hepatic vein (HVP), the portal vein (PVP) and from a peripheral vein (SVP). These blood samples were used in former studies [31–35] and there was enough material left from HVP and PVP of 38 patients and of SVP of 31 patients. Plasma was also collected shortly after TIPS, and HVP of 37 patients, PVP of 38 patients and SVP of 38 patients were available. Serum of 26 of these patients was accessible. Laboratory parameters were provided by the local Institute of Clinical Chemistry and Laboratory Medicine. Analysis of cholesteryl ester and sphingolipid species in our patients with liver cirrhosis has been done before and data were published [36]. This study revealed a negative association of circulating cholesterol with the MELD score [36].

The control cohort included patients referred to ultrasound imaging (Table 1). Patients with cancers, severe liver diseases or liver cirrhosis were excluded. Serum but not plasma was collected from these patients (Table 1).

Tumor-adjacent liver tissues of patients undergoing surgery because of hepatocellular carcinoma were used for immunoblot analysis. These patients were described in detail in a previous work, which was published in an open access journal and data are therefore freely accessible [37].

### Measurement of lipids

Analysis of lipids was recently described in detail [36]. Quantification used direct flow injection electrospray ionization tandem mass spectrometry [38, 39]. Not naturally occurring lipid species served as internal standards. Dissolved samples (10 mM ammonium acetate in methanol/chloroform (v/v)) were injected with a HTS PAL autosampler (Zwingen, Switzerland). A flow gradient (55 μl/min for 0.1 min, 30 μl/min for 1.0 min and an increase to 250 μl/min (0.2 min)) was used. The triple quadrupole mass spectrometer has an electrospray ion source which operates in positive mode (Quattro Ultima, Micromass, Manchester, UK).

A fragment ion of *m/z* 184 was used for sphingomyelin (SM), and of m/z 264 for ceramides [40]. Cholesteryl ester (CE) species were quantified by using a fragment of m/z 369, and CE 17:0/ 22:0 and deuterated D (7)-free cholesterol were used as internal standards [38].

### PCSK9 ELISA

The human PCSK9 DuoSet ELISA was used (R&D Systems; Wiesbaden, Nordenstadt, Germany) as specified by the provider (dilution 1:100). Resistin, galectin-3, IL-6, adiponectin, leptin, chemerin and pentraxin 3 were measured in serum / plasma before [31–35].

### SDS-polyacrylamide gel electrophoresis and immunoblotting

These techniques were described [37]. For quantification of signals ImageJ software was used [41]. C-reactive protein (CRP) and LDL-receptor antibodies were from R&D Systems. PCSK9 antibody and alpha-smooth muscle actin antibodies (alpha-SMA) were from Cell Signalling Technology (Danvers, MA, USA).

### Statistics

Data are visualized as box blot charts. The circles in the figures represent outliers greater than 1.5 times, and the asterisks outliers greater than 3.0 times the interquartile range. Statistical tests used were Mann-Whitney U Test, one-way ANOVA and Spearman correlations (IBM SPSS Statistics 25.0). The chi square test was used for categorical variables. A *P* value < 0.05 was considered significant.

## Results

### PCSK9 in serum of patients with and without liver cirrhosis

**Fig. 1:**
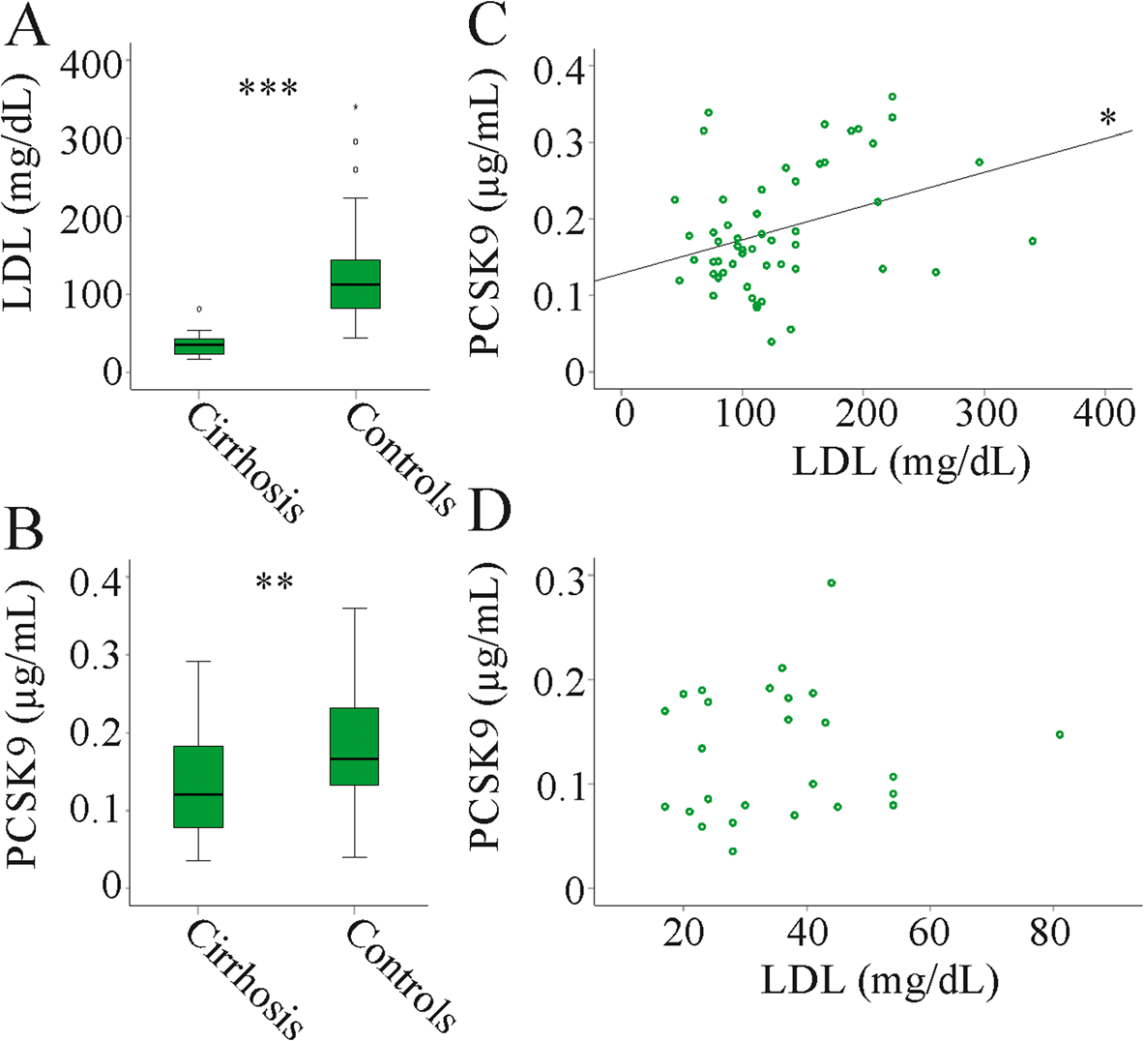
PCSK9 and LDL in serum of patients with and without liver cirrhosis. **a** LDL in serum of patients with and without liver cirrhosis (Controls). **b** PCSK9 in serum of patients with and without liver cirrhosis (Controls). **c** Correlation of PCSK9 and LDL in the control cohort. **d** Correlation of PCSK9 and LDL in the cirrhosis cohort. * *P* < 0.05

LDL is low in patients with liver cirrhosis [8] and was also reduced in the serum of the 26 patients with liver cirrhosis participating in the current study in comparison to the 55 non-cirrhotic patients (Fig. 1a). PCSK9 was also low in serum of liver cirrhosis patients (Fig. 1b). Positive correlations of PCSK9 and LDL existed in the non-cirrhotic patient group but not the cirrhotic patients (Fig. 1c, d).

PCSK9 is highly expressed in the liver [29], and impaired liver function may contribute to lower PCSK9 levels in the hepatic vein of these patients. Serum of sufficient patients was not available to clarify whether hepatic PCSK9 release is indeed associated with residual liver function. Therefore, plasma was used for this analysis.

### PCSK9 in peripheral, hepatic and portal venous plasma of patients with liver cirrhosis

**Fig. 2:**
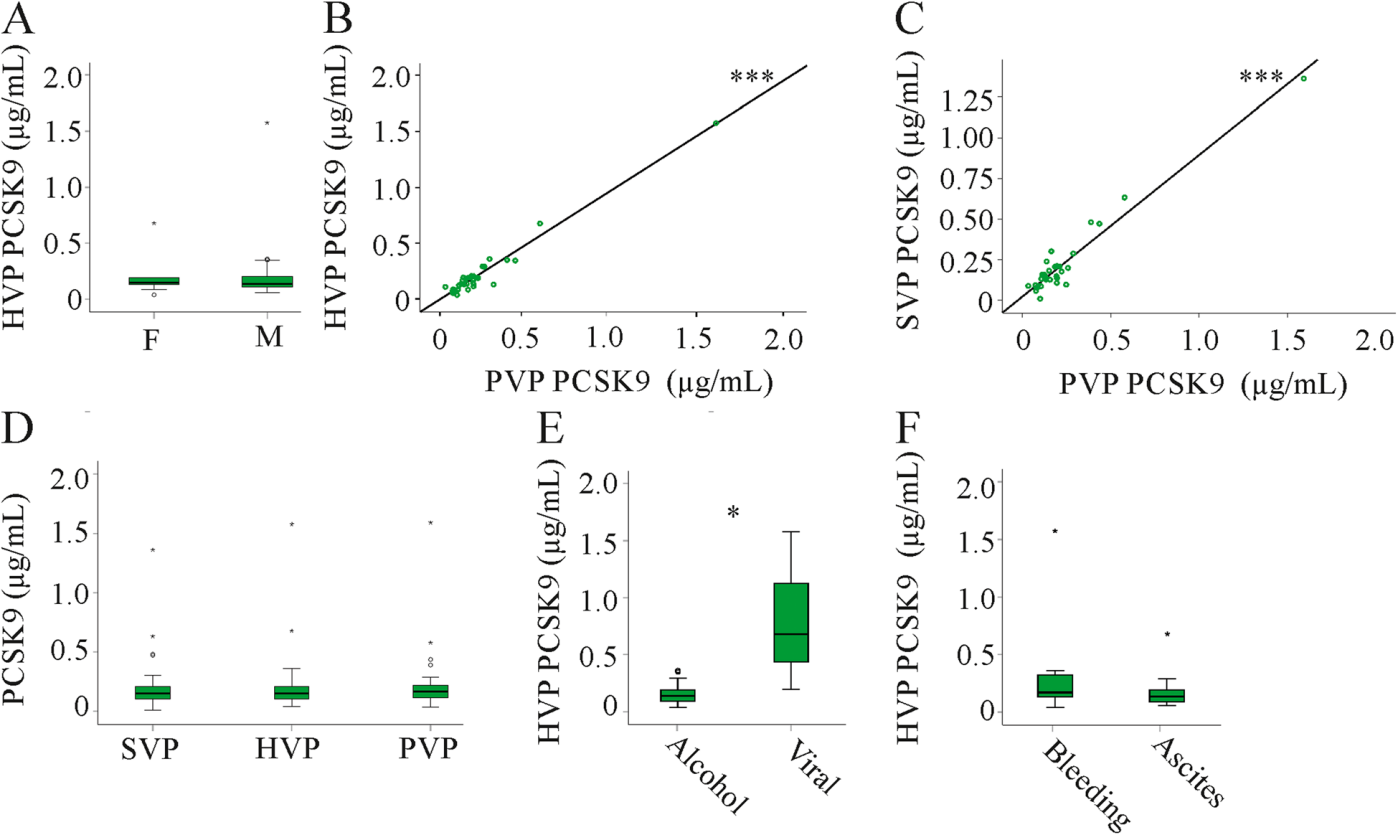
PCSK9 in plasma of patients with liver cirrhosis. **a** PCSK9 levels in females (F) and males (M). **b** Correlation of hepatic vein plasma (HVP) and portal vein plasma (PVP) PCSK9. **c** Correlation of systemic vein plasma (SVP) and PVP PCSK9. **d** PCSK9 in SVP, HVP and PVP. **e** HVP PCSK9 in 31 patients with alcoholic and 3 patients with viral disease etiology. **f** HVP PCSK9 in 12 patients and 24 patients, respectively, where variceal bleeding or ascites were the major complication. *** *p* < 0.001

Plasma of 38 patients with clinically diagnosed liver cirrhosis was available. PCSK9 was measured by ELISA in systemic vein plasma (SVP), hepatic vein plasma (HVP) and portal vein plasma (PVP). PCSK9 plasma concentrations did not differ by gender (Fig. 2a) and were not related to age (data not shown). Correlations existed for SVP and PVP PCSK9 (*r* = 0.769, *p* < 0.001), SVP and HVP PCSK9 (HVP, *r* = 0.779, *p* < 0.001) and PVP and HVP PCSK9 (*r* = 0.809, *p* < 0.001) (Fig. 2b, c and data not shown).

PCSK9 levels were comparable in SVP, HVP and PVP (Fig. 2d). The three patients with viral disease etiology had higher PCSK9 plasma levels in SVP (*P* = 0.015), HVP (Fig. 2e; *P* = 0.016) and PVP (*P* = 0.014) than patients with alcoholic liver cirrhosis. Ascites and variceal bleeding were the main complications of liver cirrhosis in the patients, but PCSK9 in HVP (Fig. 2f), SVP and PVP (data not shown) did not differ between the two cohorts.

### Association of PCSK9 with the severity and complications of liver cirrhosis

**Fig. 3:**
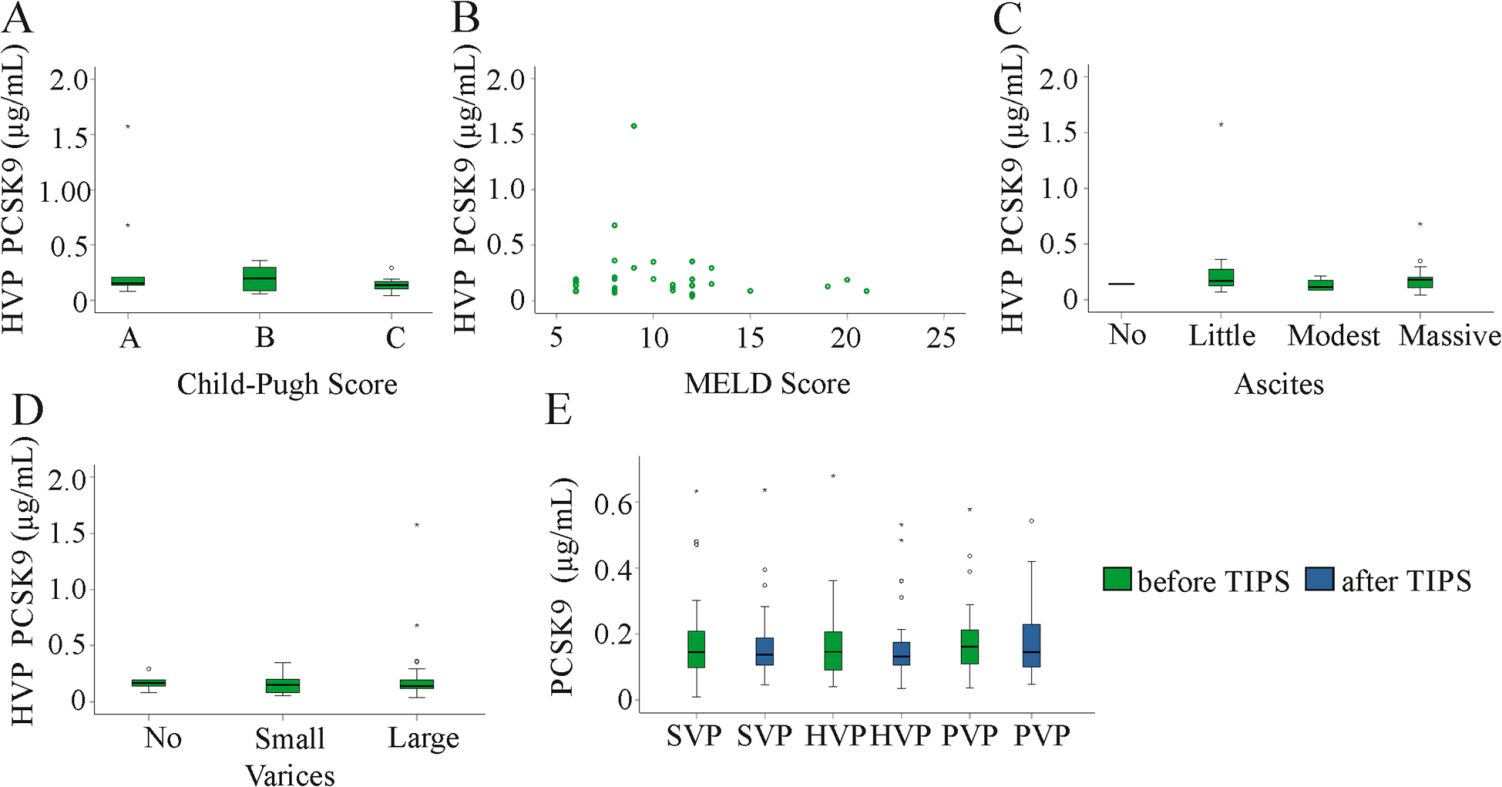
Associations of PCSK9 with Child-Pugh and MELD score and complications of liver cirrhosis. **a** PCSK9 in hepatic vein plasma (HVP) of patients stratified for Child-Pugh scores. **b** Correlation of HVP PCSK9 with the MELD score. **c** HVP PCSK9 in patients without (No), little, modest and massive ascites. **d** HVP PCSK9 in patients without, with small and large varices. **e** PCSK9 in the three compartments before and shortly after TIPS

The Child-Pugh and the MELD scores are used to assess the prognosis of liver cirrhosis [42]. No differences were observed in plasma PCSK9 levels between patients with Child-Pugh scores A, B and C (Fig. 3a and data not shown). PCSK9 levels in SVP, HVP and PVP were not correlated with the MELD score in the whole cohort and when patients with non-alcoholic disease etiology were excluded (Fig. 3b and Table 2). PCSK9 plasma concentrations did not change with ascites volume or variceal size (Fig. 3c, d). Ascites and varices are serious complications and are related to the hepatic venous pressure gradient (HVPG) [43]. HVPG of 36 patients was documented but was not correlated with PCSK9 in any blood compartment (data not shown). Transjugular intrahepatic portosystemic shunting reduced HVPG (*p* < 0.001, HVPG of 29 patients was documented) whereas PCSK9 was not changed (Fig. 3e). This finding further excluded an association of HVPG and PCSK9 levels.

**Table 2:** Correlation coefficients of PCSK9 in the different blood compartments of all patients with MELD score, bilirubin, aspartate aminotransferase (AST), alanine aminotransferase (ALT), Quick prothrombin time, antithrombin 3, fibrinogen, factor V and creatinine, which were measured in peripheral blood. Highlighted in yellow are the correlation coefficients in the subgroup of patients with alcoholic disease etiology. Superscript digits indicate number of patients where these data were available. * *P* < 0.05, ** *P* < 0.01

PCSK9	HVP	PVP	SVP
MELD	0.070 ^37^	0.079 ^38^	0.084 ^31^
	−0.126 ^31^	0.008 ^31^	0.094 ^26^
Bilirubin	−0.096 ^38^	−0.055 ^38^	− 0.018 ^31^
	−0.234 ^31^	− 0.246 ^31^	−0.119 ^26^
AST	−0.015 ^38^	0.140 ^38^	0.276 ^31^
	−0.165 ^31^	−0.043 ^31^	0.123 ^26^
ALT	−0.251 ^38^	−0.094 ^38^	− 0.029 ^31^
	−0.342 ^31^	− 0.232 ^31^	−0.086 ^26^
Albumin	−0.115 ^38^	−0.030 ^38^	− 0.476 ^31^ **
	−0.085 ^31^	0.027 ^31^	−.471 ^26^ *
Quick Prothrombin Time	0.223 ^38^	0.181 ^38^	0.277 ^31^
	0.179 ^31^	0.167 ^31^	0.317 ^26^
Antithrombin 3	0.277 ^34^	0.312 ^34^	0.308 ^27^
	0.303 ^29^	0.367 ^29^	0.350 ^24^
Fibrinogen	0.134 ^37^	0.127 ^37^	0.224 ^30^
	0.215 ^30^	0.215 ^30^	0.336 ^25^
Factor V	0.023 ^25^	0.064 ^25^	0.169 ^20^
	−0.087 ^22^	−0.046 ^22^	0.020 ^18^
Creatinine	0.002 ^38^	0.098 ^38^	0.078 ^31^
	0.048 ^31^	0.130 ^31^	0.173 ^26^

### Hepatic PCSK9 and LDL-receptor protein in liver cirrhosis

**Fig. 4:**
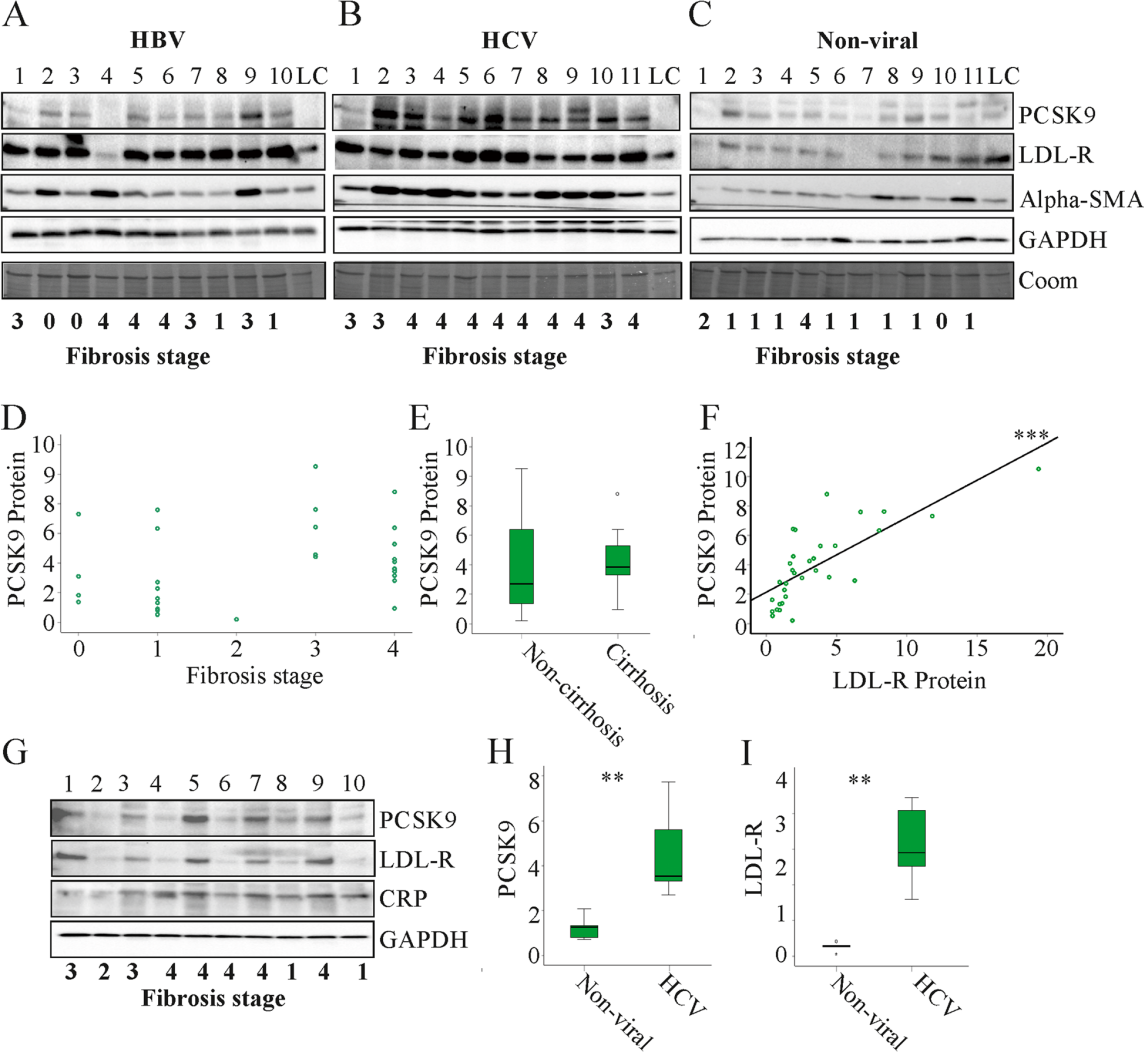
PCSK9 and LDL-receptor protein in the liver of patients with chronic liver diseases. PCSK9, LDL-receptor, alpha-SMA and GAPDH protein in the liver of patients with **a** chronic HBV infection, **b** chronic HCV infection and **c** non-viral liver disease. The respective fibrosis stages are listed below the figure panels. LC is the loading control and was used for normalization of the different immunoblots. **d** Correlation of hepatic PCSK9 protein with fibrosis stage. **e** PCSK9 in the liver of patients with fibrosis stage 0 to 3 (non-cirrhosis) and in patients with liver cirrhosis (fibrosis stage 4). **f** Correlation of hepatic PCSK9 and LDL-receptor protein. **g** PCSK9, LDL-receptor, CRP and GAPDH protein in the liver of patients with chronic HCV infection (patient number 1, 3, 5, 7, 9) and non-viral disease (patient number 2, 4, 6, 8, 10). The respective fibrosis stages are listed below the figure. **h** Quantification of PCSK9 and **i** LDL-receptor in the liver of patients with chronic HCV infection and non-viral disease. Coomassie stained membranes are shown to further confirm equal loading of the immunoblots. ** *P* < 0.01, *** *P* < 0.001

PCSK9 is expressed in the liver and murine data showed that the liver predominately contributed to serum PCSK9 levels [29]. Having shown that plasma PCSK9 did not decline with measures of liver disease severity it was suggested that hepatic PCSK9 expression may also be unaffected. PCSK9 protein was analysed by immunoblot in the liver of patients with chronic liver diseases. Liver cirrhosis was defined by histology, and 3 of the 10 patients with hepatitis B had cirrhosis. In patients with chronic HCV infection 8 of the 11 patients had liver cirrhosis and in patients with non-viral liver disease 1 of the 11 patients had liver cirrhosis. Alpha-smooth muscle actin (alpha-SMA) is induced in liver cirrhosis and, as expected, was positively correlated with fibrosis stage (Fig. 4a - c; *r* = 0.392, *P* = 0.026). PCSK9 (*r* = 0.316, *P* = 0.083) and the LDL-receptor protein (*r* = 0.174, *P* = 0.340) did not correlate with fibrosis stage and were not reduced in the patients with liver cirrhosis when compared to patients with fibrosis stage 0 to 3 (Fig. 4a - e and data not shown). Of note, hepatic PCSK9 protein positively correlated with hepatic LDL-receptor protein (Fig. 4f).

HCV infected patients had higher plasma PCSK9 (Fig. 2e) and comparison of PCSK9 protein in the liver of five patients infected with HCV and five patients with non-viral liver disease revealed that PCSK9 was induced in HCV (Fig. 4g, h). LDL-receptor protein was also higher in the liver of HCV patients (Fig. 4i). C-reactive protein (CRP) was not changed in the liver of the HCV patients, excluding that protein levels were generally higher in HCV liver (Fig. 4g).

### Correlation of plasma PCSK9 with laboratory parameters of hepatic and kidney function

PCSK9 levels did not correlate with bilirubin, Quick prothrombin time, aspartate aminotransferase, alanine aminotransferase, fibrinogen, antithrombin 3, factor V or creatinine in any blood compartment (Table 2). Albumin negatively correlated with SVP PCSK9 whereas HVP and PVP PCSK9 levels were not related to albumin (Table 2). These associations persisted when the patients with non-alcoholic disease etiology were excluded from the calculation (Table 2).

### Associations with circulating inflammatory markers and adipokines

PCSK9 is upregulated by inflammatory mediators [10]. PCSK9 in any of the three blood compartments did, however, not correlate with CRP (Table 3). Moreover, there were no associations of PCSK9 with the immune-regulatory proteins galectin-3 and resistin [20, 44, 45] (Table 3). Pentraxin 3 is an adequate indicator of inflammation in patients with liver cirrhosis [46] but was not related to PCSK9 levels in any of the blood compartments (Table 3). Moreover, PCSK9 did not correlate with interleukin-6 (IL-6) in SVP, HVP or PVP (Table 3).

**Table 3:** Correlation coefficients of PCSK9 in the different blood compartments with CRP measured in serum, and resistin, galectin-3, pentraxin 3, chemerin, adiponectin, leptin and IL-6 measured in the respective blood compartments. Highlighted in yellow are the correlation coefficients in the subgroup of patients with alcoholic disease etiology. Superscript numbers indicate the number of patients where these data were available. * *P* < 0.05, ** *P* < 0.01

PCSK9	HVP	PVP	SVP
CRP (peripheral)	0.076 ^35^	0.203 ^35^	0.308 ^30^
	−0.047 ^29^	0.081 ^29^	0.225 ^25^
Resistin	0.017 ^34^	0.074 ^34^	−0.008 ^29^
	−0.045 ^28^	0.036 ^28^	−0.046 ^24^
Galectin-3	−0.118 ^34^	0.262 ^34^	0.181 ^29^
	−0.363^28^	0.096 ^28^	−0.029 ^24^
Pentraxin 3	−0.080 ^34^	0.008 ^34^	−0.034 ^26^
	−0.030 ^30^	0.093 ^29^	0.104 ^23^
Chemerin	0.427 ^38^**	0.327 ^38^	0.300 ^31^
	0.365 ^31^*	0.222 ^31^	0.218 ^26^
Adiponectin	−0.367 ^34^*	−0.204 ^34^	−0.107 ^29^
	−0.397 ^28*^	−0.215 ^28^	− 0.080 ^24^
Leptin	0.246 ^34^	0.068 ^34^	0.212 ^29^
	0.229 ^28^	- 0.073 ^28^	0.187 ^24^
IL-6	−0.107 ^20^	− 0.244 ^20^	0.212 ^20^
	−0.232 ^16^	−0.382 ^16^	0.203 ^13^

Chemerin is highly expressed in hepatocytes and correlates with circulating markers of inflammation in distinct patient cohorts [47–49]. HVP chemerin was indeed positively associated with hepatic vein PCSK9 (Table 3). Adiponectin and leptin are adipokines with a function in liver fibrosis [20]. In HVP there was a modest, inverse correlation of PCSK9 and adiponectin (Table 3). Associations of PCSK9 with adiponectin and chemerin were still significant in the subgroup of patients with alcoholic disease etiology (Table 3). Leptin was not related to PCSK9 concentrations in any of the blood compartments (Table 3).

### Associations of plasma PCSK9 with cholesterol and sphingolipids

**Fig. 5:**
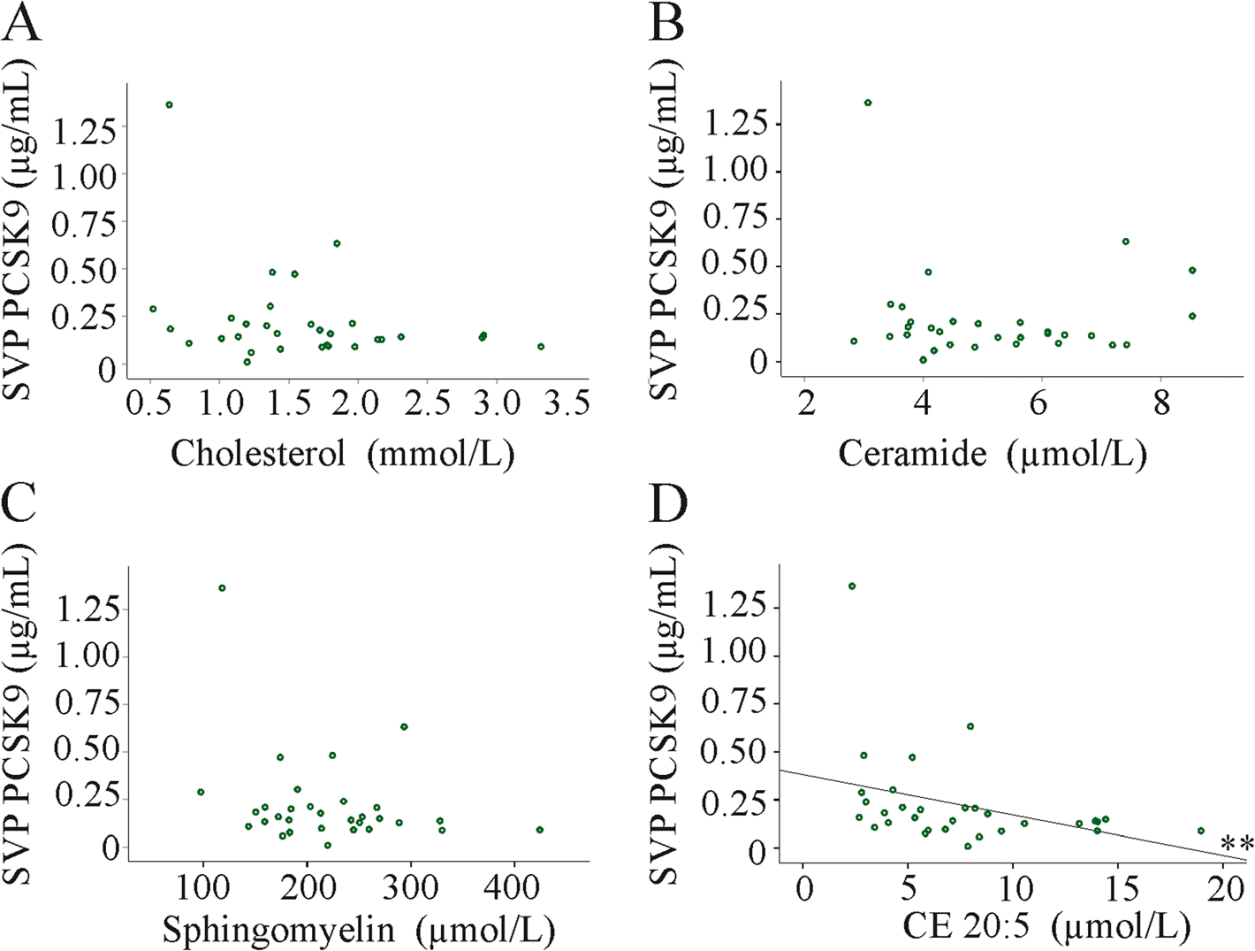
Associations of PCSK9 with cholesterol and sphingolipids in patients with liver cirrhosis. **a** Correlation of systemic vein plasma (SVP) PCSK9 with cholesterol (which is the sum of all cholesteryl esters and free cholesterol). **b** Correlation of SVP PCSK9 with ceramide. **c** Correlation of SVP PCSK9 with sphingomyelin (**d**) Correlation of SVP PCSK9 with cholesteryl ester (CE) 20:5. ** *P* < 0.01

Previous studies identified correlations between PCSK9, total and LDL cholesterol [10, 11, 28, 50]. Levels of free cholesterol, cholesteryl ester and sphingolipid species were determined in the serum of our patient cohort and these data were already published. This cohort initially included 45 patients (plasma of 7 patients was used up), and a negative correlation of cholesterol with the MELD score existed [36]. PCSK9 did not correlate with total cholesterol (which is the sum of all cholesteryl esters and free cholesterol) (*r* = − 0.282, *p* = 0.13), the sum of the cholesteryl ester levels (*r* = − 0.28, *p* = 0.13) or free cholesterol (*r* = − 0.279, *p* = 0.14) (Fig. 5a and data not shown). Moreover, PCSK9 did not correlate with total ceramide or sphingomyelin levels (Fig. 5b, c). Associations with individual ceramide or sphingolipid species could not be identified (data not shown). Referring to individual cholesteryl ester species there was a negative correlation of PCSK9 with CE 20:5 (Fig. 5d).

### Serum cholesterol and sphingolipids in patients with high and low PCSK9

**Fig. 6:**
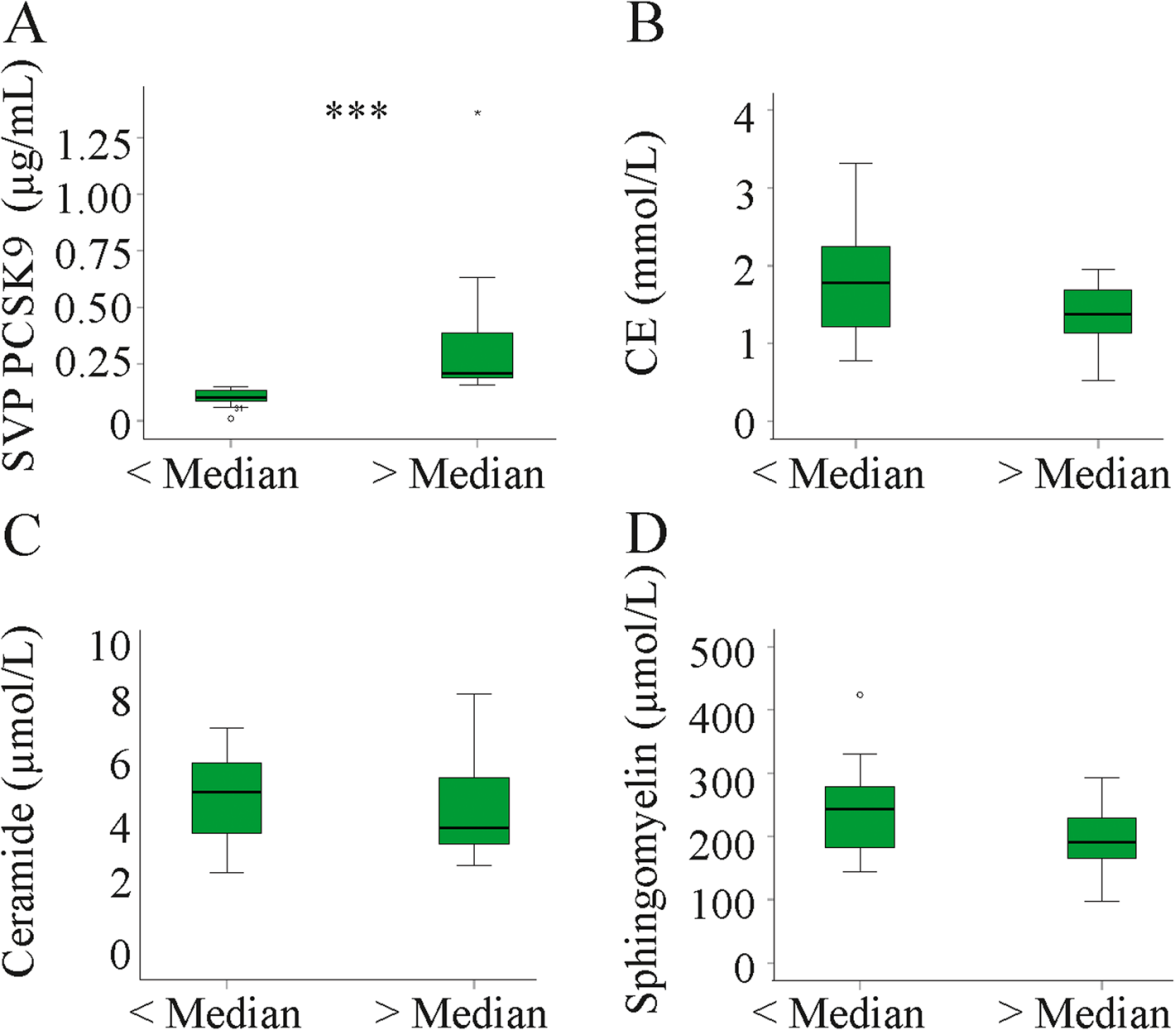
PCSK9 and lipids in patients with PCSK9 levels below or above the median plasma value. **a** PCSK9 in patients with PCSK9 below or above the median value. **b** Cholesteryl ester (CE) levels in patients with PCSK9 below or above the median value. **c** Ceramide in patients with PCSK9 below or above the median value. **d** Sphingomyelin in patients with PCSK9 below or above the median value. *** *P* < 0.001

High PCSK9 is usually associated with high cholesterol and the negative association with CE 20:5 was not expected. To investigate this in more detail the patient cohort was classified into two study groups according to the median SVP PCSK9 concentration (148.4 ng/mL), i.e. 16 patients with PCSK9 below the median and 15 patients with levels higher than the median (Fig. 6a and Table 4). These two groups had comparable age, MELD score, ALT, AST, bilirubin, CRP, resistin, galectin-3, IL-6, pentraxin 3, adiponectin and chemerin levels (data not shown). Albumin (*p* = 0.001) was lower in the group with high PCSK9. Total cholesteryl ester levels tended to be lower (*p* = 0.05, Fig. 6b). CE 16:0, 20:5, 20:4, 22:6 and 22:4 were indeed significantly diminished in the patients with higher PCSK9 (Table 4). Total ceramide and sphingomyelin (SM) levels were essentially the same in both subgroups (Fig. 6c, d). SM 18:0, 20:1, 24:2 and 24:1 were nevertheless lower in the group with high PCSK9 (Table 5).

**Table 4:** Median, minimum and maximum values of cholesteryl ester (CE) species in patients with PCSK9 levels below and above the median level. *P < 0.05, ** *P* < 0.01

PCSK9 (ng/mL) and CE species (μmol/L)	SVP PCSK9 below the median level (16 patients)			SVP PCSK9 above the median level (15 patients)			***P***-value
	Median	Min	Max	Median	Min	Max	
PCSK9	101.7	9.4	148.2	211.1	156.8	1362.3	**
CE 14:0	15.3	7.0	28.6	15.3	5.2	25.0	
CE 15:0	5.1	2.3	11.8	4.4	1.6	7.6	
CE 16:1	110.3	43.8	213.5	94.5	37.3	200.5	
CE16:0	256.4	100.4	418.9	177.7	71.4	285.5	*
CE18:3	21.5	12.2	61.6	21.4	6.5	40.5	
CE 18:2	839.7	385.5	1817.4	611.3	248.6	1200.1	
CE 18:1	381.2	168.1	672.9	307.3	105.2	452.8	
CE 18:0	5.6	3.1	21.7	6.1	2.3	13.8	
CE 20:5	8.9	3.4	18.9	4.7	2.3	8.8	**
CE 20:4	123.3	34.9	214.4	54.9	23.1	102.9	**
CE 20:3	12.1	3.5	35.5	8.8	3.5	15.6	
CE 20:2	0.9	0.4	2.9	0.7	0.0	1.6	
CE 20:1	0.6	0.3	2.8	0.5	0.2	1.9	
CE 20:0	0.8	0.5	1.5	0.7	0.6	1.1	
CE 22:6	6.4	2.1	14.8	3.9	1.9	8.9	**
CE 22:5	1.3	0.3	2.9	0.9	0.5	2.6	
CE 22:4	0.5	0.2	1.6	0.4	0.0	1.3	*
CE 22:1	0.4	0.2	1.4	0.3	0.0	0.7

**Table 5:** Median, minimum (Min) and maximum (Max) values of serum sphingomyelin (SM) species in patients with PCSK9 levels below and above the median level. * *P* < 0.05

SM species (μmol/L)	SVP PCSK9 below the median level (16 patients)			SVP PCSK9 above the median level (15 patients)		***P***-value	
	Median	Min	Max	Median	Min	Max	
SM 14:0	4.9	2.7	9.0	4.4	2.0	5.8	
SM 15:0	2.9	1.5	4.7	2.3	0.9	3.9	
SM16:1	11.2	5.7	22.3	8.0	4.7	16.6	
SM16:0	69.2	40.3	126.6	58.6	26.3	86.3	
SM 18:2	0.2	0.0	0.9	0.3	0.0	0.6	
SM 18:1	5.8	2.4	12.2	3.6	1.8	9.2	
SM 18:0	9.8	3.8	18.7	6.2	0.0	12.4	*
SM 20:1	3.0	1.4	5.3	2.3	1.4	3.6	*
SM 20:0	6.1	2.2	9.8	6.9	2.6	11.3	
SM 22:2	2.1	0.0	3.9	2.1	0.0	4.5	
SM 22:1	12.8	6.8	21.4	10.3	0.0	18.6	
SM 22:0	11.2	5.2	20.3	9.2	4.9	16.1	
SM 23:1	5.9	2.5	12.3	4.5	2.8	9.0	
SM 23:0	3.7	1.1	8.8	2.9	1.7	6.2	
SM 24:3	1.6	0.0	3.6	1.5	0.3	4.3	
SM 24:2	20.9	10.8	36.6	15.9	10.7	24.2	*
SM 24:1	50.1	36.1	95.0	41.8	18.4	71.2	*
SM 24:0	7.1	4.7	13.6	6.6	3.3	10.9	
SM 26:2	0.5	0.0	1.0	0.3	0.0	1.1	
SM 26:1	0.5	0.0	1.1	0.3	0.0	0.8	

## Discussion

In patients with liver cirrhosis the present study demonstrated that plasma PCSK9 was neither associated with the severity of liver cirrhosis nor its complications. PCSK9 did not positively correlate with serum cholesterol and sphingolipids, which are carried in LDL particles. In fact, in patients with liver cirrhosis, impaired hepatic function seems to override the effect of PCSK9 on cholesterol metabolism, as demonstrated by the loss of the correlation between cholesterol and PCSK9. Actually, distinct cholesteryl ester species even declined in patients with PCSK9 levels above the median concentration in comparison to patients with PCSK9 levels below the median. Though the mechanisms of these associations have not been finally resolved, this finding excludes a role of PCSK9 in alcoholic cirrhosis related hypocholesterolemia. Of note, a positive correlation of PCSK9 and LDL-receptor protein existed in the liver of patients with chronic liver diseases. Thus, PCSK9 mediated degradation of the LDL-receptor protein is hindered in the diseased liver by a so far unknown mechanism.

A lack of correlation between cholesterol and PCSK9 was also reported in a cohort of patients with severe liver disease where the MELD score ranged from 7 to 40, with a mean score of 21 [24]. This study described a negative association of PCSK9 and the MELD score and a higher mortality of patients with low PCSK9 [24]. It could be suggested that impaired hepatic synthesis of PCSK9 is a consequence of the severely disturbed liver function of patients with very high MELD scores [24]. Noteworthy, PCSK9 correlated with parameters of liver function like albumin supporting this assumption [24]. The MELD score of our patients ranged from 6 to 21 and, in contrast to the study described above, was not related to PCSK9 levels. In patients with PCSK9 above the median value albumin was lower in comparison to patients with PCSK9 levels below the median. Moreover, analysis of the association between PSCK9 and albumin revealed a significant negative correlation. Thus, our findings do not support an association of circulating PCSK9 and residual liver function at least in patients with alcoholic liver cirrhosis. A separate study observed higher hepatic PCSK9 protein in the cirrhotic compared to the non-cirrhotic liver [25]. Bhat et al. further described a significant positive correlation of hepatic PCSK9 protein with the stages of liver fibrosis [25]. In that study 85% of patients had liver cirrhosis, and only 6 patients had fibrosis stage 0, 2 or 3 [25]. In the tissues analysed herein 12 patients had cirrhosis and 20 patients had fibrosis stage 0, 1, 2 or 3, and therefore, the cohort may be better suited to identify associations of hepatic PCSK9 protein and stages of liver fibrosis.

Analysis of hepatic PCSK9 protein in patients with viral and non-viral disease etiology failed to identify higher levels in the cirrhotic liver. Similarly, PCSK9 protein did not change with increasing liver disease severity in patients with NAFLD [12].

So far it is unclear whether the etiology of liver cirrhosis has an effect on circulating PCSK9. Comparison of hepatic PCSK9 protein in patients with viral and non-viral related liver diseases showed that PCSK9 protein was strongly induced in HCV liver. In line with a previous study, LDL-receptor protein was also high in the liver of HCV patients [51]. The LDL-receptor is involved in hepatitis C virus (HCV) entry, and PCSK9 was supposed to protect from HCV infection [52]. The three patients with HCV cirrhosis had higher plasma PCSK9 than patients with alcoholic etiology. Indeed, increased and reduced PCSK9 levels were observed in HCV patients [53] and HCV genotype or comorbidities may be potential confounding factors.

In the study by Schlegel et al. showing correlations of PCSK9 with the MELD score the underlying etiologies varied and 62% of the patients had alcoholic liver diseases. The other patients had non-alcoholic fatty liver disease, HCV, cryptogenic cirrhosis, Budd-Chiari syndrome and autoimmune hepatitis [24]. Separate analysis of the patients with alcoholic liver disease was not performed [24] adding to the differences between the data of the present examination and the study by Schlegel et al. [24].

Median PCSK9 was 148 ng/mL in our patient cohort and 106 ng/mL in the cohort with very severe liver diseases [24]. PCSK9 concentrations described in the literature range from about 50 to 600 ng/mL and may vary according to the underlying disease [10, 24, 25, 54–56]. The high variation of PCSK9 in the different studies further suggests that absolute concentrations may vary depending on the type of ELISA assay used. It is not recommended to compare PCSK9 levels determined by different assays. Therefore, we measured PCSK9 in serum of non-cirrhotic controls and patients with liver cirrhosis. Serum PCSK9 was lower in the patients with liver cirrhosis. Positive associations of PCSK9 and LDL existed in the non-cirrhotic patients. This association was lost in liver cirrhosis patients. In the cohort analysed by Bhat et al. serum PCSK9 was 92 ng/mL in the 6 healthy controls and 78 ng/mL in the 13 cirrhosis patients, also suggesting a decline of circulating PCSK9 in liver cirrhosis [25].

Most ELISAs for PCSK9 measurement can not distinguish between intact PCSK9 and its truncated, inactive form [57]. This latter isoform is produced by furin cleavage and represents 3 to 42% of systemic PCSK9 [57]. Correlation coefficients with LDL-cholesterol did not improve when concentrations of intact PCSK9 instead of total PCSK9 levels were used for calculation [57]. So far levels of intact and cleaved PCSK9 were not determined in liver cirrhosis patients. The missing correlation of circulating PCSK9 with cholesterol may be also caused by a high level of inactive PCSK9 in patients with liver cirrhosis, a suggestion which has to be proven in the future.

Discordant findings regarding the association of circulating PCSK9 and liver function [24, 25] suggest that additional factors affect circulating PCSK9. PCSK9 is highly expressed in the intestine and small intestinal bacterial overgrowth is common in patients with liver cirrhosis [58, 59]. LPS induced hepatic PCSK9 but whether intestinal PCSK9 production was affected by LPS or was associated with liver disease severity was not evaluated in that study [59]. Intestinal PCSK9 did not regulate plasma lipids and did not contribute to PCSK9 plasma levels [60]. Accordingly, the current analysis showed that PCSK9 concentrations did not differ between the portal and the hepatic vein.

Systemic inflammation contributes to the progression of liver cirrhosis [61] and LPS increased hepatic PCSK9 expression in mice [62]. Therefore, associations of PCSK9 with circulating inflammatory proteins were analysed in our cohort. CRP is an acute phase protein and is produced in the liver in response to IL-6 [20]. Pentraxin 3 is expressed by vascular smooth muscle cells and endothelial cells at the site of inflammation and is an independent inflammatory biomarker [46]. Analysis of the association between PCSK9, IL-6 and CRP or pentraxin 3 revealed no significant correlations. This also applied for resistin and galectin-3, which are both induced in activated immune cells [20].

In the cohort studied in the current work, PCSK9 was negatively correlated with adiponectin and positively with chemerin in the hepatic vein. Adiponectin is regarded as an anti-inflammatory and antifibrotic adipokine [63]. The role of chemerin in the liver was less well analysed but several studies identified positive correlations of systemic chemerin and inflammatory cytokines [47, 49]. Recombinant chemerin added to culture media did not regulate PCSK9 in hepatocellular carcinoma cell lines [64] and there is no evidence that PCSK9 affects hepatocyte chemerin expression. Serum chemerin is positively and adiponectin is negatively associated with an unfavourable metabolic phenotype in a population-based cohort study [65]. Such associations may change in severely ill patients including patients with liver cirrhosis [20]. Serum adiponectin was induced in liver cirrhosis patients and this may be a consequence of impaired hepatic excretion [20]. On the other hand, chemerin declined in parallel with the severity of liver disease [66]. This suggests that associations of PCSK9 and the adipokines chemerin and adiponectin in the hepatic vein possibly reflect the activity of certain pathways which are impaired in liver cirrhosis.

The liver is the major source of plasma PCSK9 at least in mice [29] and hepatic LDL-receptors are major regulators of PCSK9 clearance [67]. Similar PCSK9 concentrations in the hepatic and the portal vein suggest that hepatic synthesis and clearance of PCSK9 must be equal. The half-life of PCSK9 in blood is about 5 min [67] and hepatic synthesis seems to compensate for loss of blood PCSK9.

### Study strengths and limitations

The strength of this study is that plasma collected from the portal and the hepatic vein of patients with liver cirrhosis was available. Moreover, individual cholesteryl ester and sphingolipid species rather than total levels were measured. Limitations of the present analysis were that the number of patients enrolled was rather small and that the exact time points of blood sampling were not documented. Moreover, possible confounding factors like medications and nutritional status were not considered. Liver tissues analysed were from patients with viral and non-viral disease etiologies but not from patients with alcoholic liver cirrhosis, and findings may not be valid for these patients. Future studies should also discriminate between lipids carried in LDL, HDL and VLDL particles.

## Conclusions

The present examinations indicate that the negative association between the hepatic LDL-receptor protein levels and PCSK9 was lost in patients with severe liver diseases. Indeed, levels of these proteins were positively correlated in the liver. Accordingly, distinct cholesteryl ester species were even increased in patients with low plasma PCSK9. Identification of the underlying mechanisms is essential to clarify whether blockage of PCSK9 may be a suitable approach to reduce LDL levels in patients with severe liver fibrosis.

## Data Availability

The datasets generated and/or analysed during the current study are available from the corresponding author on reasonable request.

## Abbreviations

ALT: Alanine aminotransferase
Alpha-SMA: Alpha-Smooth Muscle Actin
AST: Aspartate aminotransferase
CRP: C-reactive protein
CE: Cholesteryl ester
HDL: High density lipoprotein
HVP: Hepatic vein plasma
INR: International normalized ratio
LDL: Low density lipoprotein
LPS: Lipopolysaccharide
MELD: Model for End Stage Liver Disease
NASH: Non-alcoholic steatohepatitis
PCSK9: Proprotein convertase subtilisin/kexin type 9
PVP: Portal vein plasma
SM: Sphingomyelin
SVP: Systemic vein plasma
TIPS: Transjugular intrahepatic portosystemic shunt
